# The influence of parental awareness of the “Chinese double reduction” policy on junior high school students’ extracurricular physical exercise

**DOI:** 10.3389/fpsyg.2023.1079523

**Published:** 2023-03-02

**Authors:** Ping Liu, Jin Chen, Yangyang Shen, Hu Lou

**Affiliations:** School of Sports Science, Nantong University, Nantong, China

**Keywords:** double reduction, educational anxiety, exercise attitude, physical exercise, parent

## Abstract

**Objective:**

In this study, we aimed to explore the relationship between parental cognitive awareness (criticality, disruption and novelty cognition) of DBR, educational anxiety, and attitudes toward students’ physical exercise and students’ extracurricular physical exercise so as to construct and verify a conditional process model.

**Methods:**

We adopted a stratified random cluster sampling approach and conducted a nationwide questionnaire survey with 2,700 junior high school students and their parents across 9 provinces, municipalities and autonomous regions.

**Results:**

Parents generally had a certain degree of cognitive awareness of DBR. Criticality cognition and disruption cognition had a significant positive impact on junior high school students’ extracurricular physical exercise (*β* = 0.24, *p* < 0.01; *β* = 0.04, *p* < 0.05), but the effect of novelty cognition was not significant (*β* = −0.04, *p* = 0.06). Parents’ educational anxiety played a significant mediating role in parents’ cognition of DBR and students’ extracurricular physical exercise (criticality cognition: *β* = 0.10, 95% CI: 0.03–0.06; disruption cognition: *β* = 0.37, 95% CI: 0.31–0.42). Parents’ attitude toward students’ exercise also played a significant positive moderating effect in the mediation model (criticality cognition: *β* = 0.014, 95% CI: 0.002–0.031; disruption cognition: *β* = 0.010, 95% CI: 0.007–0.013).

**Conclusion:**

Parents’ novelty, disruption, and criticality cognition of DBR have different effects on parents’ education anxiety and students’ extracurricular physical exercise, in which parental educational anxiety mediates the influence of DBR cognition on students’ extracurricular physical exercise, while attitudes toward students’ extracurricular physical exercise positively moderates the mediating effect.

## 1. Introduction

On July 24, 2021, the Chinese government issued a statement on “the opinions on further reducing academic burden of students in compulsory education and the burden of off-campus training,” hereinafter referred to as DBR, which aims to promote the all-round development and healthy growth of students (Ma and Zheng, 2022). DBR is intended to provide more time and energy for students to participate in extracurricular physical exercise. Researchers in the field of sports have concluded that students’ extracurricular physical activities will provide new opportunities for rapid development (Chai et al., 2022). Historically, the problems of students’ academic burden have been long-standing and a series of “burden reduction” policies have been introduced in the history of education in New China (Li and Zhao, 2022). However, despite the promulgation and implementation of these policies, the academic burden of primary and secondary school students persists. Moreover, the problem of low level of extracurricular physical activities has become increasingly serious. It is therefore important to evaluate the concrete effects of DBR in the first year of its inception. The purpose of this study was to analyze the effects of DBR on the extracurricular physical exercise of junior high school students after it was enacted, and to explore the role of parents’ educational anxiety and their attitudes toward their children’s participation in extracurricular physical exercise.

## 2. Literature review and research hypothesis

### 2.1. Evaluations of parental cognition of DBR and junior high school students’ extracurricular physical exercise based on policy performance

Performance evaluation of government public policies is currently a much-discussed topic in the area of administrative research, and is vital for improving the effectiveness of public policies (He et al., 2021). The impact of such policies is often evaluated based on expected targets and empirical data (Zhao et al., 2021), and is carried out both prior to and following the end of policy implementation activities. It further focuses on the assessment and improvement of possible effects from the perspective of policy objectives. Due to complex dynamics of society, government public policies often empirically demonstrate the response effects of policy implementation from a systemic point view of. If the implementation of DBR is considered as an event in the social system, then the event system theory can be used to test a series of impacts of the policy (Morgeson et al., 2015). Event system theory claims that the attributes of time, space, and intensity of events require special attention to explain the effect of events on individuals (Zhang and Yan, 2018).

Since this study concerned the practical impact of DBR on middle school students over the past year, variables such as time (policy timing, policy duration) and space (policy origin, diffusion range, diffusion distance) are relatively steady in the cross-sectional survey of individual event responses, and so can be reflected separately by the intensity of the event (Cheng and Yin, 2021). The intensity of the event comprises the three dimensions of novelty, disruption, and criticality to measure the effect of the policy (Morgeson and Derue, 2006). *Novelty* reflects the degree to which the policy differs from current or past policies, and represents a new and unexpected phenomenon or event. *Disruption* reflects the extent to which policy changes organizations or individuals. *Criticality* reflects the significance of a policy to an organization or individual and the priority level of response. In summary, this study mainly measures the intensity of events from individual cognitions of the novelty, disruption, and criticality of DBR in order to explore the impact of the policy on junior high school students’ extracurricular physical exercise.

Extracurricular physical activities have been shown to enrich students’ out-of-class life, strengthen physique, enhance personality, and promote mental health (Lou and Yan, 2020; Yan et al., 2020; Li and Kwok, 2021), it is an important content to reflect the effect of DBR (Pei et al., 2022). Moreover, DBR is directed at the compulsory education stage, where students are relatively young and parents’ perceptions are crucial to the effectiveness of the policy (Zhao and Fan, 2022). Therefore, this study focuses on parental cognition of the policy as the primary independent variable of the model. As mentioned above, DBR provides a new opportunity to improve the extracurricular physical activity for junior high school students. Combining with the event system theory, this study proposes the following hypotheses:

*H1a*: Parental scores on novelty cognition of DBR have a significant positive effect on students’ participation in extracurricular physical exercise.

*H1b*: Parental scores on disruption cognition of DBR have a significant positive effect on students’ participation in extracurricular physical exercise.

*H1c*: Parental scores on criticality cognition of DBR have a significant positive effect on students’ participation in extracurricular physical exercise.

### 2.2. The mediating role of parents’ educational anxiety

DBR aims to both ease the burden on students and promote healthy development, as well as reduce the burden and pressure on parents. According to a survey conducted by the Central Committee of the Communist Youth League, the educational anxiety of students’ parents in compulsory education has been alleviated after the implementation of DBR (CNTV, 2021). Moreover, event system theory suggests that when events occur, individuals will have cognitive and emotional reactions to them. Parents’ educational anxiety is not only a widespread negative emotion among members of society, but also has a profound influence on adolescents’ learning, life, and health, marking it as an important phenomenon for Chinese parents (Jin, 2015). Relevant studies have also shown that parents’ educational anxiety is detrimental to their children’s extracurricular physical exercise (Wei, 2021). Based on this, we further propose the following hypotheses:

*H2a*: Parental scores on novelty cognition of DBR have a significant negative influence on parents’ educational anxiety.

*H2b*: Parental scores on disruption cognition of DBR have a significant negative influence on parents’ educational anxiety.

*H2c*: Parental scores on criticality cognition of DBR have a significant negative influence on parents’ educational anxiety.

*H2d*: Parental scores on educational anxiety have significant negative influence on students’ extracurricular physical exercise.

In addition, parental scores on cognition of the event intensity of DBR (novelty cognition, disruption cognition and criticality cognition) affect junior high school students’ extracurricular physical activity, which may be achieved through the intermediary of parents’ educational anxiety. Thus, we predict that parental awareness of the event intensity of DBR possibly reduces parents’ educational anxiety. Subsequently, the reduction of parents’ educational anxiety may increase the extracurricular physical activity level of junior high school students. We further propose that:

*H3a*: Parents’ educational anxiety plays a mediating role between parents’ novelty cognition of DBR and junior high school students’ extracurricular physical exercise.

*H3b*: Parents’ educational anxiety plays a mediating role between parents’ disruption cognition of DBR and junior high school students’ extracurricular physical exercise.

*H3c*: Parents’ educational anxiety plays a mediating role between parents’ criticality cognition of DBR and junior high school students’ extracurricular physical exercise.

### 2.3. The moderating role of parents’ attitudes toward their children’s participation in physical exercise

Teenagers in their formative years find that their behaviors are easily influenced by others. As the most important influencers of junior high school students, parents exert a subtle effect on their extracurricular physical activities (Milliken, 2021; Niu and Wang, 2021). There is a positive correlation among parents’ attitudes toward their children’s sports participation and sports behaviors (Anderson et al., 2009; Chen et al., 2021). Parents’ positive attitudes toward their children’s participation in extracurricular physical activity has the ability to amplify the impact of reduced parents’ educational anxiety on middle-school students’ extracurricular physical activity. In contrast, if the level of parents’ attitudes is low, the relief of parents’ educational anxiety may not bring students a higher level of extracurricular physical exercise. Consequently, we hypothesize that:

*H4a*: Parents’ attitudes toward their children’s participation in extracurricular physical exercise can significantly and positively affect junior high school students’ extracurricular physical exercise.

*H4b*: Parents’ attitudes toward their children’s participation in extracurricular physical exercise play a moderating role between parents’ educational anxiety and junior high school students’ extracurricular physical exercise.

**Figure 1:**
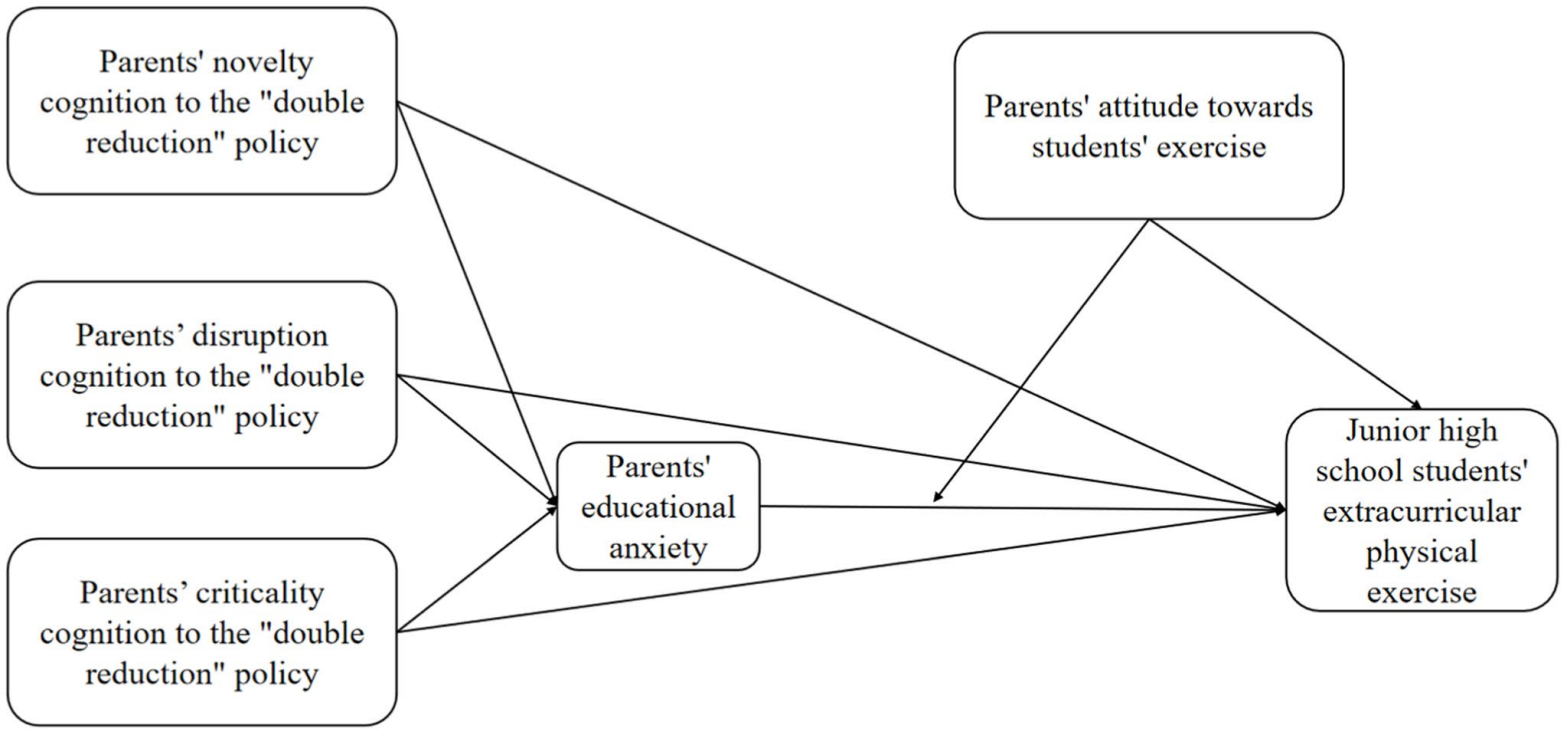
Hypothetical model of parents’ cognition of DBR impact on junior high school students’ extracurricular physical exercise.

A full hypothetical model depicting these predictions is shown in Figure 1.

Based on the above schematic, we explore the effects and pathways of parents’ policy cognition scores on junior high school students’ extracurricular physical exercise in the context of DBR. In addition, we investigate the role of parents’ educational anxiety in parents’ cognition intensity of DBR (novelty cognition, disruption cognition, and criticality cognition) and the effect of this anxiety on middle school students’ extracurricular physical activity, in addition to the moderating role of parents’ attitudes toward their children’s participation in extracurricular physical exercise.

## 3. Research methods

### 3.1. Research objects

We adopted the method of stratified random cluster sampling to comprehensively consider the three major economic belts in the east, middle, and west of China. We selected three low-grade cities: in the east: Nantong, Jiangsu Province, Lishui, Zhejiang Province, and Fuxin, Liaoning Province; in the middle: Taiyuan, Shanxi Province, Puyang, Henan Province, and Xiangxi Tujia and Miao Autonomous Prefecture, Hunan Province; in the west: Chongqing, Yibin, Sichuan Province, and Yili, Xinjiang Uygur Autonomous Region. From March to April 2022, local teachers were instructed to distribute 100 paper questionnaires in each grade of 9 junior middle schools, 2,700 of which were distributed and 2,679 were recovered, including 2,420 valid questionnaires (recovery rate = 89.6%). The valid data included 1,190 male students and 1,230 female students with a basically balanced sex ratio. Of these, 826 students were in Grade 7, 913 students were in Grade 8, and 681 students were in Grade 9 (grade distribution: *X^2^* = 3.74, *p* = 0.15).

### 3.2. Measuring tools

#### 3.2.1. Parents’ cognition of DBR

We administered the Chinese version of the Event Intensity Cognition Scale revised in combination with DBR (Liu and Liu, 2017). There are 11 items in total, including 4 reverse scoring items, using the Likert 7-point scoring method, in which 1 represents “completely disagree” and 7 represents “completely agree.” Higher scores indicate higher parental cognition of DBR. Four of the items reflect novelty cognition, three items reflect criticality cognition, and the final four items reflect disruption cognition of DBR. The dimensions show high reliability (McDonald ω = 0.911, 0.911, and 0.912, respectively). Confirmatory factor analysis (*X^2^/df* = 4.924 < 5, RMSEA = 0.041 < 0.08, NFI = 0.982 > 0.9, IFI = 0.985 > 0.9, TLI = 0.980 > 0.9, CFI = 0.985 > 0.9) further confirm high structural validity.

#### 3.2.2. Parents’ educational anxiety

We used the Parents’ Education Anxiety Questionnaire (Han, 2018) containing 21 items in total on a Likert 5-point scale. Higher scores indicate higher parental anxiety about their children’s education, and the scale includes dimensions of anxiety about children’s learning attitudes, school choice, future development, academic achievements, and anxiety about parent–child learning interaction. The dimensions show high reliability (Cronbach’s α = 0.785, 0.782, 0.809, 0.811 and 0.849 respectively), and confirmatory factor analyses (*X^2^/df* = 4.410 < 5, RMSEA = 0.038 < 0.08, NFI = 0.974 > 0.9, IFI = 0.980 > 0.9, TLI = 0.975 > 0.9, CFI = 0.980 > 0.9) indicate good structural validity.

#### 3.2.3. Parents’ attitude toward their children’s participation in extracurricular physical exercises

We used the Parental Sports Attitude Scale revised in combination with the factor of extracurricular physical exercise (Bao and Teng, 2001). The Likert scale includes 9 total items, and higher scores indicate more positive parental attitudes toward students’ extracurricular physical exercise. The dimensions show high reliability (Cronbach’s α = 0.921), and good structural validity (*X^2^/df =* 4.097 < 5, RMSEA = 0.036 < 0.08, NFI = 0.992 > 0.9, IFI = 0.994 > 0.9, TLI = 0.992 > 0.9, CFI = 0.994 > 0.9).

#### 3.2.4. Extracurricular physical exercise for junior high school students

We final used the Physical Activity Rating Scale revised in combination with the dimension of “extracurricular physical exercise” (Liang, 1994). The scale includes 3 total items combining frequency of weekly extracurricular exercise, duration of each extracurricular exercise, and the intensity of ordinary extracurricular exercise; the total score of was the product of the three. The answer to each item is divided into five grades, with a scoring range of frequency and intensity of 1–5, a scoring range of time of 0–4, and the total score range of 0–100. Exercise intensity dimension: “1” representing “Slight intensity,” “5” representing “Continuous high intensity.” Exercise frequency dimension: “1” representing “Once a month,” “5” representing “Once a day.” Each duration dimension: “0” representing “Less than 10 min,” “4” representing “More than 60 min.” Higher scores indicate higher levels of students’ extracurricular physical exercise. The internal consistency coefficient of the scale was Cronbach’s α = 0.907.

### 3.3. Common method biases test

Data were collected using the self-report method. In order to detect the possibility of common method bias, we used the Harman single factor test prior to data analysis in which all variables were analyzed using non-rotated principal component analysis. The results showed that the variance explained by the first factor was 22.86%, less than the critical value of 40%. Therefore, there is no significant evidence for common method bias in the data.

### 3.4. Data analysis

SPSS25.0 was used for descriptive statistics and correlation analysis of variables (see Tables 1, 2), and linear regression analysis was used for main effect test (see Table 3). Process 4.0 plug-in is used to analyze the mediation effect and regulatory effect. Model 4 was used to test the mediating effect of parents’ educational anxiety (see Table 4), and model 59 was used to test the moderating effect of parents’ attitudes (see Table 5). The bootstrap method based on 2,000 resample was used to examine the significance of the direct and indirect effects.

**Table 1:** Basic information of extracurricular physical exercise of junior high school students.

PARS	*N*	%
**Frequency**		
Once a month	120	5
2–3 times a month	934	39
1–2 times a week	753	31
3–5 times a week	517	21
Once a day	96	4
**Intensity**		
Slight intensity	321	13
Minimum intensity	414	17
Medium intensity	939	39
High intensity	480	20
Continuous high intensity	266	11
**Duration**		
Less than 10 min	527	22
11–20 min	706	29
21–30 min	768	32
31–59 min	266	11
More than 60 min	153	6

**Table 2:** Descriptive statistics and correlation analysis results of cognition variables.

	Variables	M	SD	1	2	3	4	5	6	7	8
1	Gender	-	-	1							
2	Stage	-	-	−0.003	1						
3	Novelty cognition	2.870	1.167	−0.020	−0.011	1					
4	Criticality cognition	3.136	1.248	0.037	−0.018	−0.084**	1				
5	Disruption cognition	3.262	1.236	−0.025	−0.065*	−0.150**	0.295**	1			
6	Educational anxiety	2.910	0.721	−0.011	0.054**	0.077**	−0.265**	−0.356**	1		
7	Parents’ attitude	2.886	0.960	0.021	−0.011	−0.127**	0.066**	0.232**	−0.063**	1	
8	PARS	8.670	6.859	0.010	0.040	−0.080**	0.53**	0.265**	−0.150**	0.092**	1

*p < 0.05, **p < 0.01.

**Table 3:** Main effects hypothesis test.

Variables	Parents’ educational anxiety			PARS		
	*β*	*SE*	*p*	*β*	*SE*	*p*
Novelty cognition	0.01	0.05	0.36	−0.04	0.02	0.06
Disruption cognition	−0.30**	0.05	0.00	0.24**	0.03	0.00
Criticality cognition	−0.18**	0.06	0.00	0.04*	0.03	0.04
Educational anxiety				−0.07**	0.01	0.01
Parents’ attitude				0.03	0.01	0.14
*R^2^*	0.16					
*F*	147.80					

*p < 0.05, **p < 0.01.

**Table 4:** Bootstrap test results of mediation effects.

Effect type	*β*	SE	LLCI	ULCI
**Independent variables: Novelty cognition**				
Total effect	−0.12	0.03	−0.17	−0.06
Direct effects	−0.10	0.03	−0.16	−0.04
Indirect effects	−0.02	0.01	−0.03	0.01
**Independent variables: Disruptive cognition**				
Total effect	0.37	0.03	0.31	0.42
Direct effects	0.33	0.03	0.28	0.39
Indirect effects	0.03	0.01	0.01	0.06
**Independent variables: Critical cognition**				
Total effect	0.10	0.04	0.02	0.17
Direct effects	0.03	0.04	−0.05	0.10
Indirect effects	0.07	0.01	0.05	0.09

**Table 5:** Mediated moderating effects of parents’ educational anxiety.

Paths	*β*	SE	LLCI	ULCI
**Moderator variables: Parents’ attitudes toward their children’s exercise**				
Novelty cognition → parental educational anxiety → extracurricular physical exercise	0.006	0.001	−0.004	0.009
Disruption cognition → parental educational anxiety → extracurricular physical exercise	0.010	0.001	0.007	0.013
Criticality cognitive → parental educational anxiety → extracurricular physical exercise	0.014	0.008	0.002	0.031

## 4. Results

### 4.1. Descriptive statistics and correlation analysis

The descriptive statistics (Table 1) show that most junior high school students have participated in extracurricular physical exercise. Parents’ cognition of DBR scores are shown in Table 2.

The correlations between PARS and the various cognition variables are shown in Table 2.

### 4.2. Main effects test

We found that novelty cognition scores had no significant impact on students’ extracurricular physical exercise (*β* = −0.04, *SE* = 0.02, *p* = 0.06), in contrast to *H1a*. We also did not find evidence for the effect of novelty cognition on parents’ educational anxiety (*β* = 0.01, *SE* = 0.05, *p* = 0.36). However, disruption cognition did have a significant positive effect on students’ extracurricular physical exercise (*β* = 0.24, *SE* = 0.03, *p* < 0.01), consistent with our prediction *H1b*. We also found evidence in favor of the remaining hypotheses: criticality cognition had a significant positive effect on students’ extracurricular physical exercise (*β* = 0.04, *SE* = 0.03, *p* < 0.05); disruption cognition had a significant negative effect on parents’ educational anxiety (*β* = −0.30, *SE* = 0.05, *p* < 0.01), and criticality cognition had a significant negative impact on parents’ educational anxiety (*β* = −0.18, *SE* = 0.06, *p* < 0.01). Furthermore, parents’ educational anxiety showed a significant negative effect on students’ extracurricular physical exercise (*β* = −0.07, *SE* = 0.01, *p* < 0.05). Parents’ attitudes toward their children’s participation in physical exercise had a positive but nonsignificant impact on junior high school students’ extracurricular physical exercise (*β* = 0.03, *SE* = 0.01, *p* = 0.14; Table 3).

### 4.3. Mediating and moderating effects testing

The mediating effects of the test results are shown in Table 4. When novelty cognition is entered as the independent variable, there is a negative total effect on students’ extracurricular physical exercise (*β* = −0.12, 95%CI = [−0.17, −0.06]) in which the negative direct effect is significant (*β* = −0.10, 95%CI = [−0.16, −0.04]), but not the indirect effect (*β* = −0.02, 95%CI = [−0.03, 0.01]), thus not supporting our *H3a* prediction. When considering disruption cognition as the independent variable, the total effect on students’ extracurricular physical exercise is significant (*β* = 0.37, 95%CI = [0.31, 0.42]), and both the direct (*β* = 0.33, 95%CI = [0.28, 0.39]) and indirect effects (*β* = 0.03, 95%CI = [0.01, 0.06]) are significant, suggesting that parental educational anxiety plays a partial mediation role. Finally, using criticality cognition as the independent variable results in a total significant effect on students’ extracurricular physical exercise (*β* = 0.10, 95%CI = [0.03, 0.06]), while the direct effect is not significant (*β* = 0.02, 95%CI = [−0.05, 0.10]), but the indirect effect is (*β* = 0.07, 95%CI = [0.05, 0.09]). These results suggest that parental educational anxiety plays a full mediating role, supporting prediction *H3c*.

**Figure 2:**
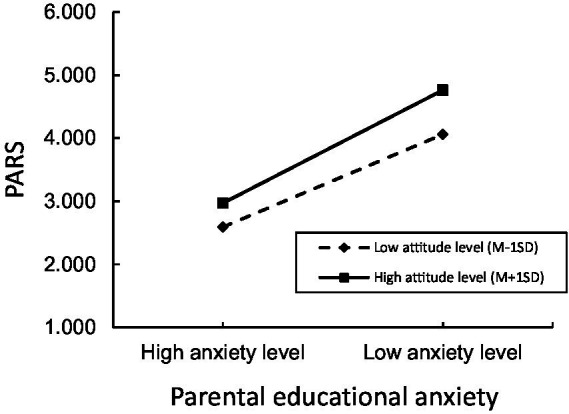
The moderating effect of parents on children’s exercise attitudes.

The interaction between parents’ educational anxiety and attitudes toward their children’s participation in extracurricular physical exercise shows a significant positive effect on junior high school students’ participation in extracurricular physical exercise (*β* = 0.007, *SE* = 0.001, *p* < 0.01). Figure 2 shows the results of a simple slops test depicting the differences between “high” and “low” groupings (designated as one standard deviation above and below the mean, respectively). The results show that the effect of parental educational anxiety on students’ extracurricular physical exercise is stronger under the condition of high parental exercise attitude and weaker in conditions of low parental exercise attitudes.

We further tested the mediating and moderating role of parents’ attitude toward their children’s participation in extracurricular physical exercise (Table 5). The results show that parental educational anxiety is a significant mediated moderating variable in all aspects of cognition.

## 5. Discussion

### 5.1. Parents’ policy cognition, educational anxiety, and junior high school students’ extracurricular physical exercise in the context of DBR

Our results show that parents of junior high school students are generally aware of the promulgation and the impact of DBR (cognitive intensity mean range: 2.870–3.262). However, the frequency of extracurricular physical activity among junior high school students has remained steady, as shown by the low percentage of those who never participate in extracurricular physical activity and the high percentage of those who participate three times a week or more, differing slightly different from the results of studies prior to the outbreak of COVID-19 (Yan et al., 2020). The reason for this may be that the enactment of DBR has had a greater impact on society, schools, families, and individuals, and that teachers, parents, and junior high school students are all increasingly aware of the importance of healthy development and need for extracurricular physical activity. This is consistent with social-ecological theory models (Lee and Park, 2021), in which the policy layer at the top of the model can influence extracurricular physical activity of society, schools, interpersonal, and individual junior high school students at the macro level. Given the effectiveness of encouraging and promoting youth physical activity through policy adjustments, the investigation of DBR in this study becomes especially relevant and crucial.

The educational anxiety levels of parents in different dimensions vary in scale with respect to each other. For example, parental anxiety about parent–child interactions is seemingly less significant than anxiety about adolescents’ academic performance, which is in turn less important than anxiety about adolescents’ learning attitudes, future development, and adolescents’ choice of school for higher education. This is consistent with findings obtained prior to DBR (Han, 2018), in which parent–child interaction anxiety was lowest, but is not consistent with Han’s findings about anxiety about children’s learning attitudes. This may be because under DBR, parents are increasingly aware of the importance of the healthy growth of adolescents in junior high school and their anxiety about their children’s studies has been alleviated. However, at the present stage in China, entry into high school is selected and classified according to the results of the secondary school entrance examination; therefore, anxiety about school selection remains a critical component of parental educational anxiety. Our findings show that the enactment of DBR may have had an impact on alleviating parental anxiety and promoting extracurricular physical activity in adolescents.

### 5.2. Effects of parents’ cognition of DBR on junior high school students’ extracurricular physical activity

First, we explored the effects of the three dimensions of parents’ cognition of DBR (novelty cognition, disruption cognition, and criticality cognition) on junior high school students’ extracurricular physical activity. The results showed that parents’ novelty cognition of DBR did not have a significant effect on junior high school students’ extracurricular physical activity, while disruption and critical cognition had a significant positive effect on extracurricular physical activity. Since junior high school students are mainly only children or two-child families, parents may not have comparative experience with the implementation of the previous reduction policy, leading to decreased relevance and awareness of the novelty cognition aspect of DBR. Moreover, the novelty aspect may not directly affect students’ physical activity outside of class. It is also possible that novelty may create a degree of uncertainty for parents who may be hesitant to allow students to participate in extracurricular physical activity because the consequences may be unclear.

The significance of criticality cognition may be due to the fact that parents’ awareness of reducing the burden on students and promoting the healthy development of their children is currently a priority in the education field and may consequently facilitate the reduction of extracurricular classes and homework time and increase the amount of healthy extracurricular physical activity. Furthermore, if parents recognize that DBR is critical to their children’s future success, they may also be less likely to promote more extracurricular physical activity by reducing the need for quick fixes. Finally, the disruption cognition results may be a result of parents believing that the implementation of DBR will disrupt the current status quo in family education and require changes to previously established educational practices to promote behaviors (e.g., extracurricular physical activity) that are beneficial to their children’s physical and mental health. Although there is little empirical research to support the idea that students’ extracurricular physical activity will increase after DBR, some studies (Wang et al., 2022; Zhang et al., 2022) argue that DBR should bring opportunities to increase students’ extracurricular physical activity, which is consistent with the findings we obtained here.

### 5.3. Mediating and moderating effects of parents’ educational anxiety and attitudes toward their Children’s exercise

Parents’ novelty cognition of DBR had a positive effect on parents’ educational anxiety but did not reach statistical significance, suggesting that the relationship between novelty cognition and anxiety may be more complex than initially thought. We hypothesized that parents’ perception of the novelty of DBR would reduce parents’ educational anxiety, but the results did not support this hypothesis. This may be because if parents perceive the policy as a novelty, they may develop new anxieties due to their lack of understanding of the new policy, thereby presenting a more complex situation. Studies on parents’ educational anxiety in China also suggest that parents’ uncertainty in the educational process and educational outcomes can induce educational anxiety, which can manifest itself in complex emotional states such as tension, anxiety, apprehension, and annoyance (Chen and Xiao, 2014). In contrast, parents’ disruption and criticality cognition scores negatively influenced educational anxiety. Therefore, if parents are aware of the importance of DBR in the current educational system, they may experience lower educational anxiety, which comes with a fuller comprehension of the policy.

We further found that parents’ educational anxiety negatively affects students’ extracurricular physical activity, consistent with prior research (Song and Feng, 2021). Chinese parents’ educational anxiety is obvious and even called “severe anxiety disorder.” Parents always want their children to be better than other children, so the phenomenon of over-education is serious, and children’s learning burden is becoming heavier and heavier, without following the laws of education and the physical and mental development characteristics of adolescent. Only by reducing this anxiety can they untie their children and let them participate in activities that are beneficial to their healthy growth and development.

Parents’ educational anxiety plays a partially mediating role in the process of parents’ disruption cognition of DBR on students’ extracurricular physical exercise, and plays a completely mediating role in the impact of criticality cognition on students’ extracurricular physical exercise. This can further explain the mechanism and characteristics of the effect of DBR on the extracurricular physical activity of junior high school students. The implementation of DBR may have alleviated parents’ educational anxiety and relieved students from the burden of subject tutoring or homework, which in turn facilitated more extracurricular physical activity.

Parents’ attitudes toward their children’s participation in extracurricular physical activity under DBR did not significantly affect junior high school students’ extracurricular physical activity, perhaps due to the fact that parents received information about DBR that required additional cognitive processing. If parents only know that DBR has been implemented but do not recognize the criticality and disruptive nature of its content, this would not effectively reduce their anxiety nor promote extracurricular physical activity among their children. The moderating effects obtained in this study further reinforces the view that parents’ attitudes toward their children’s participation in extracurricular physical activity can positively moderate the effect of educational anxiety on students’ extracurricular physical activity. In other words, students’ extracurricular physical activity is more likely to occur under the dual effect of reduced parents’ educational anxiety and positive parental attitudes toward their participation in extracurricular physical activity. Moreover, the role of parental educational anxiety alleviation in promoting junior high school students’ extracurricular physical activity was strongest under conditions of high levels of parental attitudes and weaker under conditions of low levels of attitudes.

## 6. Conclusion

We demonstrated that parents’ cognitive awareness of the novelty, disruption and criticality dimensions of DBR have differing effects on their education anxiety and their children’s participation in extracurricular physical exercise. Moreover, we found that parents’ educational anxiety mediates the influence of these DBR cognition aspects on students’ extracurricular physical exercise, while parental attitudes toward students’ extracurricular physical exercise positively regulates the mediating effect. In the future, it is expected to further promote the extracurricular physical exercise of Chinese junior high school students by reducing parents’ educational anxiety and cultivating parents’ attitudes towards their children’s exercise.

## Data availability statement

The raw data supporting the conclusions of this article will be made available by the authors, without undue reservation.

## Ethics statement

The studies involving human participants were reviewed and approved by Ethics Committee of Nantong University. Written informed consent to participate in this study was provided by the participants’ legal guardian/next of kin.

## Author contributions

PL and HL: study concept and design and critical revision of the manuscript for important intellectual content. JC and YS: analysis and interpretation of data and statistical analysis. PL, JC, YS, and HL: drafting of the manuscript and study supervision. All authors contributed to the article and approved the submitted version.

## Funding

The study was supported by General Program of Education of the National Social Science Fund of China: “Research on sports regulation mechanism and intervention scheme of middle school students’ psychological pressure” (BLA210215).

## Conflict of interest

The authors declare that the research was conducted in the absence of any commercial or financial relationships that could be construed as a potential conflict of interest.

## Publisher’s note

All claims expressed in this article are solely those of the authors and do not necessarily represent those of their affiliated organizations, or those of the publisher, the editors and the reviewers. Any product that may be evaluated in this article, or claim that may be made by its manufacturer, is not guaranteed or endorsed by the publisher.
